# *N*-(3-Nitro­phen­yl)-*N*′-pivaloylthio­urea

**DOI:** 10.1107/S1600536808014530

**Published:** 2008-05-21

**Authors:** M. Sukeri M. Yusof, Siti Hajar Muharam, M. B. Kassim, Bohari M. Yamin

**Affiliations:** a Department of Chemical Sciences, Faculty of Science and Technology, Universiti Malaysia Terengganu, Mengabang Telipot, 21030 Kuala Terengganu, Malaysia; b School of Chemical Sciences and Food Technology, Universiti Kebangsaan Malaysia, 43600 Bangi, Selangor, Malaysia

## Abstract

In the title compound, C_12_H_15_N_3_O_3_S, there is an intra­molecular N—H⋯O hydrogen bond. The crystal structure is stabilized by inter­molecular N—H⋯O, N—H⋯S and C—H⋯S hydrogen bonds, forming a two-dimensional network parallel to the *ac* plane.

## Related literature

For related crystal structures, see: Saeed & Flörke (2007 ▶); Sultana *et al.* (2007 ▶).
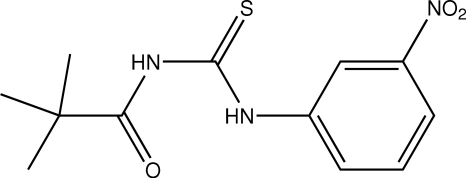


## Experimental

### 

#### Crystal data

C_12_H_15_N_3_O_3_S
*M* *_r_* = 281.33
Orthorhombic, 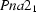

*a* = 20.400 (5) Å
*b* = 10.886 (3) Å
*c* = 6.2120 (15) Å
*V* = 1379.5 (6) Å^3^
*Z* = 4
Mo *K*α radiation
μ = 0.24 mm^−1^
*T* = 273 (2) K
0.48 × 0.18 × 0.12 mm

#### Data collection

Bruker SMART APEX CCD area-detector diffractometer
Absorption correction: multi-scan (*SADABS*; Bruker, 2000 ▶) *T*_min_ = 0.893, *T*_max_ = 0.972
8152 measured reflections
3020 independent reflections
2321 reflections with *I* > 2σ(*I*)
*R*_int_ = 0.032

#### Refinement

*R*[*F* ^2^ > 2σ(*F* ^2^)] = 0.042
*wR*(*F* ^2^) = 0.103
*S* = 0.91
3020 reflections
172 parameters
1 restraint
H-atom parameters constrained
Δρ_max_ = 0.28 e Å^−3^
Δρ_min_ = −0.14 e Å^−3^
Absolute structure: Flack (1983 ▶), 1296 Friedel pairs
Flack parameter: 0.07 (9)

### 

Data collection: *SMART* (Bruker, 2000 ▶); cell refinement: *SAINT* (Bruker, 2000 ▶); data reduction: *SAINT*; program(s) used to solve structure: *SHELXS97* (Sheldrick, 2008 ▶); program(s) used to refine structure: *SHELXL97* (Sheldrick, 2008 ▶); molecular graphics: *SHELXTL* (Sheldrick, 2008 ▶); software used to prepare material for publication: *SHELXTL*, *PARST* (Nardelli, 1995 ▶) and *PLATON* (Spek, 2003 ▶).

## Supplementary Material

Crystal structure: contains datablocks global, I. DOI: 10.1107/S1600536808014530/sg2240sup1.cif

Structure factors: contains datablocks I. DOI: 10.1107/S1600536808014530/sg2240Isup2.hkl

Additional supplementary materials:  crystallographic information; 3D view; checkCIF report

## Figures and Tables

**Table 1:** Hydrogen-bond geometry (Å, °)

*D*—H⋯*A*	*D*—H	H⋯*A*	*D*⋯*A*	*D*—H⋯*A*
N2—H2*A*⋯O1	0.86	1.92	2.605 (3)	135
N1—H1*A*⋯S1^i^	0.86	2.76	3.582 (2)	160
C3—H3*A*⋯S1^i^	0.96	2.83	3.742 (3)	159
N2—H2*A*⋯O2^ii^	0.86	2.52	3.197 (3)	137

Symmetry codes: (i) 
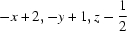
; (ii) 
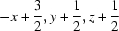
.

**Fig. 1.**
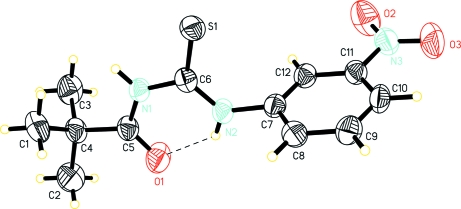
: The molecule of (I) showing the atom-labelling scheme. Displacement ellipsoids are drawn at the 50% probability level. Dashed lines indicate intramolecular hydrogen bond.

**Fig. 2.**
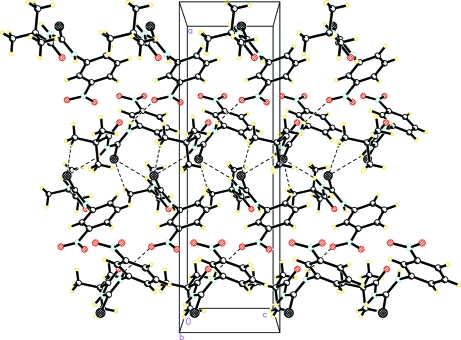
: Packing diagram of compound,(I), viewed down the b axis. The dashed lines denote the N—H···O, N—H···S and C—H···S hydrogen bonds.

## supplementary crystallographic information

### Comment

Two isomers of *N*-nitrophenyl-*N*'-pivaloylthiourea were reported by Saeed & Flörke, (2007) (nitro group at *ortho* position) and Sultana *et al.*, (2007) (nitro group at *para* position). Here, the molecule with a nitro group in the *meta* position, (I), has been successfully synthesized (Fig. 1). The molecule displays similar bond distances and angles to the related compounds.

The carbonylthiourea (S1/N1/N2/O1/C4–C7) and 3-nitrophenyl fragments are essentially planar, with maximum deviation of 0.077Å for atom O2 from the least square plane. The carbonylthiourea fragment makes a dihedral angle of 85.64 (7)° to the nitrophenyl fragment. There is an intramolecular hydrogen bond, N2—H2···O1 leading to a pseudo-six membered ring (O1···H2—N2—C6—N1—C5—O1). In the crystal structure, the molecules are linked by intermolecular interactions, N—H···O, N—H···S and C—H···S (symmetry codes as in Table 1) forming a two dimensional network along the *ac* plane (Fig.2).

### Experimental

To a stirring acetone solution (75 ml) of pivaloyl chloride (5.0 g, 0.04 mol) and ammonium thiocyanate (3.15 g, 0.04 mol), 3-nitroaniline (5.73 g, 0.04 mol) in 40 ml of acetone was added dropwise. The solution mixture was refluxed for 1 h. The resulting solution was poured into a beaker containing some ice blocks. The white precipitate was filtered off and washed with distilled water and cold ethanol before being dried under vacuum. Good quality crystals were obtained by recrystallization from THF.

### Refinement

After their location in the difference map, all H-atoms were fixed geometrically at ideal positions and allowed to ride on the parent C or N atoms with C—H = 0.93–0.97Å and N—H = 0.86Å with *U*_iso_(H)= 1.2 (CH_2_ and NH) or 1.5*U*_eq_(C)(CH_3_).

### Crystal data

**Table:**

C_12_H_15_N_3_O_3_S	*F*_000_ = 592
*M**_r_* = 281.33	*D*_x_ = 1.355 Mg m^−^^3^
Orthorhombic, *P**n**a*2_1_	Mo *K*α radiation λ = 0.71073 Å
Hall symbol: P 2c -2n	Cell parameters from 925 reflections
*a* = 20.400 (5) Å	θ = 2.0–27.5º
*b* = 10.886 (3) Å	µ = 0.24 mm^−^^1^
*c* = 6.2120 (15) Å	*T* = 273 (2) K
*V* = 1379.5 (6) Å^3^	Block, colourless
*Z* = 4	0.48 × 0.18 × 0.12 mm

### Data collection

**Table:**

Bruker SMART APEX CCD area-detector diffractometer	3020 independent reflections
Radiation source: fine-focus sealed tube	2321 reflections with *I* > 2σ(*I*)
Monochromator: graphite	*R*_int_ = 0.032
Detector resolution: 83.66 pixels mm^-1^	θ_max_ = 27.5º
*T* = 298(2) K	θ_min_ = 2.0º
ω scans	*h* = −21→26
Absorption correction: multi-scan(SADABS; Bruker, 2000)	*k* = −13→14
*T*_min_ = 0.893, *T*_max_ = 0.972	*l* = −7→7
8152 measured reflections	

### Refinement

**Table:**

Refinement on *F*^2^	Hydrogen site location: inferred from neighbouring sites
Least-squares matrix: full	H-atom parameters constrained
*R*[*F*^2^ > 2σ(*F*^2^)] = 0.042	*w* = 1/[σ^2^(*F*_o_^2^) + (0.0642*P*)^2^ + 0.0515*P*] where *P* = (*F*_o_^2^ + 2*F*_c_^2^)/3
*wR*(*F*^2^) = 0.103	(Δ/σ)_max_ < 0.001
*S* = 0.91	Δρ_max_ = 0.28 e Å^−^^3^
3020 reflections	Δρ_min_ = −0.14 e Å^−^^3^
172 parameters	Extinction correction: none
1 restraint	Absolute structure: Flack (1983), 1296 Friedel pairs
Primary atom site location: structure-invariant direct methods	Flack parameter: 0.07 (9)
Secondary atom site location: difference Fourier map	

### Special details

**Table:**

Geometry. All e.s.d.'s (except the e.s.d. in the dihedral angle between two l.s. planes) are estimated using the full covariance matrix. The cell e.s.d.'s are taken into account individually in the estimation of e.s.d.'s in distances, angles and torsion angles; correlations between e.s.d.'s in cell parameters are only used when they are defined by crystal symmetry. An approximate (isotropic) treatment of cell e.s.d.'s is used for estimating e.s.d.'s involving l.s. planes.
Refinement. Refinement of *F*^2^ against ALL reflections. The weighted *R*-factor *wR* and goodness of fit *S* are based on *F*^2^, conventional *R*-factors *R* are based on *F*, with *F* set to zero for negative *F*^2^. The threshold expression of *F*^2^ > σ(*F*^2^) is used only for calculating *R*-factors(gt) *etc*. and is not relevant to the choice of reflections for refinement. *R*-factors based on *F*^2^ are statistically about twice as large as those based on *F*, and *R*- factors based on ALL data will be even larger.

### Fractional atomic coordinates and isotropic or equivalent isotropic displacement parameters (Å^2^)

**Table:**

	*x*	*y*	*z*	*U*_iso_*/*U*_eq_	
S1	0.97040 (3)	0.39600 (5)	0.62603 (13)	0.04412 (17)	
O3	0.72971 (13)	0.0240 (2)	0.9230 (4)	0.0936 (8)	
O2	0.73374 (10)	0.13216 (17)	0.6346 (5)	0.0791 (6)	
O1	0.84682 (10)	0.73243 (15)	0.7556 (4)	0.0694 (6)	
N2	0.86872 (9)	0.49974 (16)	0.8218 (3)	0.0422 (5)	
H2A	0.8456	0.5640	0.8480	0.051*	
N1	0.92866 (9)	0.62483 (14)	0.5965 (3)	0.0394 (5)	
H1A	0.9603	0.6304	0.5055	0.047*	
C11	0.79218 (11)	0.2008 (2)	0.9299 (4)	0.0414 (5)	
C10	0.81336 (11)	0.1747 (2)	1.1350 (5)	0.0490 (6)	
H10A	0.7999	0.1035	1.2052	0.059*	
C9	0.85486 (13)	0.2563 (2)	1.2331 (5)	0.0521 (6)	
H9A	0.8704	0.2395	1.3708	0.062*	
C8	0.87392 (11)	0.36328 (19)	1.1304 (5)	0.0463 (5)	
H8A	0.9018	0.4187	1.1985	0.056*	
C7	0.85101 (11)	0.38661 (19)	0.9253 (4)	0.0387 (5)	
C12	0.80979 (11)	0.3062 (2)	0.8226 (4)	0.0413 (5)	
H12A	0.7942	0.3224	0.6848	0.050*	
N3	0.74809 (11)	0.11334 (19)	0.8218 (5)	0.0557 (6)	
C6	0.91904 (11)	0.50999 (19)	0.6876 (4)	0.0375 (5)	
C5	0.89385 (11)	0.73105 (18)	0.6338 (5)	0.0421 (5)	
C4	0.91691 (13)	0.8460 (2)	0.5166 (4)	0.0451 (6)	
C3	0.90038 (15)	0.8322 (3)	0.2772 (5)	0.0635 (8)	
H3A	0.9239	0.7634	0.2189	0.095*	
H3B	0.9128	0.9056	0.2019	0.095*	
H3C	0.8541	0.8188	0.2610	0.095*	
C2	0.87985 (15)	0.9559 (2)	0.6113 (7)	0.0733 (8)	
H2B	0.8905	0.9641	0.7611	0.110*	
H2C	0.8335	0.9430	0.5958	0.110*	
H2D	0.8923	1.0294	0.5363	0.110*	
C1	0.99039 (14)	0.8627 (2)	0.5451 (6)	0.0625 (8)	
H1B	1.0130	0.7938	0.4834	0.094*	
H1C	1.0005	0.8681	0.6957	0.094*	
H1D	1.0041	0.9368	0.4742	0.094*	

### Atomic displacement parameters (Å^2^)

**Table:**

	*U*^11^	*U*^22^	*U*^33^	*U*^12^	*U*^13^	*U*^23^
S1	0.0528 (3)	0.0349 (3)	0.0446 (3)	0.0050 (2)	0.0020 (3)	0.0025 (3)
O3	0.124 (2)	0.0680 (13)	0.0885 (16)	−0.0522 (13)	0.0132 (15)	−0.0002 (13)
O2	0.0909 (15)	0.0660 (12)	0.0804 (15)	−0.0173 (10)	−0.0336 (15)	0.0052 (14)
O1	0.0707 (13)	0.0443 (10)	0.0930 (16)	0.0126 (9)	0.0308 (12)	0.0156 (10)
N2	0.0457 (10)	0.0311 (9)	0.0497 (11)	0.0041 (8)	0.0075 (10)	0.0086 (8)
N1	0.0440 (10)	0.0325 (9)	0.0416 (13)	−0.0003 (7)	0.0044 (10)	0.0064 (8)
C11	0.0378 (12)	0.0376 (12)	0.0488 (14)	−0.0003 (10)	0.0062 (11)	0.0013 (11)
C10	0.0557 (14)	0.0387 (11)	0.0526 (14)	−0.0019 (10)	0.0128 (15)	0.0136 (15)
C9	0.0650 (16)	0.0508 (14)	0.0404 (13)	0.0030 (13)	−0.0015 (12)	0.0109 (12)
C8	0.0488 (12)	0.0427 (11)	0.0474 (13)	−0.0016 (9)	−0.0003 (15)	−0.0004 (14)
C7	0.0396 (13)	0.0366 (12)	0.0399 (13)	0.0020 (9)	0.0051 (10)	0.0043 (10)
C12	0.0423 (13)	0.0409 (12)	0.0409 (13)	0.0024 (10)	0.0016 (11)	0.0056 (11)
N3	0.0540 (13)	0.0435 (12)	0.0695 (16)	−0.0055 (10)	0.0068 (12)	−0.0009 (11)
C6	0.0391 (11)	0.0367 (12)	0.0366 (14)	−0.0034 (10)	−0.0084 (9)	0.0029 (9)
C5	0.0441 (12)	0.0350 (10)	0.0471 (12)	0.0004 (9)	0.0006 (13)	0.0067 (13)
C4	0.0520 (15)	0.0313 (11)	0.0519 (15)	−0.0003 (11)	0.0008 (12)	0.0078 (10)
C3	0.0761 (19)	0.0570 (16)	0.0575 (19)	−0.0034 (14)	−0.0070 (15)	0.0183 (14)
C2	0.093 (2)	0.0359 (12)	0.091 (2)	0.0162 (13)	0.020 (2)	0.0097 (18)
C1	0.0643 (18)	0.0414 (14)	0.082 (2)	−0.0118 (13)	−0.0026 (15)	0.0018 (13)

### Geometric parameters (Å, °)

**Table:**

S1—C6	1.668 (2)	C8—C7	1.381 (4)
O3—N3	1.217 (3)	C8—H8A	0.9300
O2—N3	1.216 (3)	C7—C12	1.371 (3)
O1—C5	1.222 (3)	C12—H12A	0.9300
N2—C6	1.327 (3)	C5—C4	1.523 (3)
N2—C7	1.435 (3)	C4—C1	1.520 (4)
N2—H2A	0.8600	C4—C3	1.532 (4)
N1—C5	1.377 (3)	C4—C2	1.532 (4)
N1—C6	1.386 (3)	C3—H3A	0.9600
N1—H1A	0.8600	C3—H3B	0.9600
C11—C10	1.375 (4)	C3—H3C	0.9600
C11—C12	1.375 (3)	C2—H2B	0.9600
C11—N3	1.472 (3)	C2—H2C	0.9600
C10—C9	1.370 (4)	C2—H2D	0.9600
C10—H10A	0.9300	C1—H1B	0.9600
C9—C8	1.384 (3)	C1—H1C	0.9600
C9—H9A	0.9300	C1—H1D	0.9600
			
C6—N2—C7	123.23 (18)	N1—C6—S1	119.22 (17)
C6—N2—H2A	118.4	O1—C5—N1	121.3 (2)
C7—N2—H2A	118.4	O1—C5—C4	121.9 (2)
C5—N1—C6	128.0 (2)	N1—C5—C4	116.8 (2)
C5—N1—H1A	116.0	C1—C4—C5	110.3 (2)
C6—N1—H1A	116.0	C1—C4—C3	110.0 (2)
C10—C11—C12	122.7 (2)	C5—C4—C3	108.4 (2)
C10—C11—N3	118.8 (2)	C1—C4—C2	110.4 (2)
C12—C11—N3	118.6 (2)	C5—C4—C2	107.8 (2)
C9—C10—C11	118.2 (2)	C3—C4—C2	109.9 (3)
C9—C10—H10A	120.9	C4—C3—H3A	109.5
C11—C10—H10A	120.9	C4—C3—H3B	109.5
C10—C9—C8	120.9 (3)	H3A—C3—H3B	109.5
C10—C9—H9A	119.5	C4—C3—H3C	109.5
C8—C9—H9A	119.5	H3A—C3—H3C	109.5
C7—C8—C9	119.0 (2)	H3B—C3—H3C	109.5
C7—C8—H8A	120.5	C4—C2—H2B	109.5
C9—C8—H8A	120.5	C4—C2—H2C	109.5
C12—C7—C8	121.3 (2)	H2B—C2—H2C	109.5
C12—C7—N2	119.6 (2)	C4—C2—H2D	109.5
C8—C7—N2	119.1 (2)	H2B—C2—H2D	109.5
C7—C12—C11	117.9 (2)	H2C—C2—H2D	109.5
C7—C12—H12A	121.1	C4—C1—H1B	109.5
C11—C12—H12A	121.1	C4—C1—H1C	109.5
O2—N3—O3	123.7 (3)	H1B—C1—H1C	109.5
O2—N3—C11	118.3 (2)	C4—C1—H1D	109.5
O3—N3—C11	118.0 (3)	H1B—C1—H1D	109.5
N2—C6—N1	116.21 (19)	H1C—C1—H1D	109.5
N2—C6—S1	124.57 (17)		
			
C12—C11—C10—C9	−1.5 (4)	C10—C11—N3—O3	3.4 (3)
N3—C11—C10—C9	179.4 (2)	C12—C11—N3—O3	−175.7 (2)
C11—C10—C9—C8	1.2 (4)	C7—N2—C6—N1	178.4 (2)
C10—C9—C8—C7	−0.6 (4)	C7—N2—C6—S1	−2.2 (3)
C9—C8—C7—C12	0.2 (4)	C5—N1—C6—N2	2.6 (4)
C9—C8—C7—N2	177.6 (2)	C5—N1—C6—S1	−176.9 (2)
C6—N2—C7—C12	−87.3 (3)	C6—N1—C5—O1	−2.1 (4)
C6—N2—C7—C8	95.2 (3)	C6—N1—C5—C4	177.8 (2)
C8—C7—C12—C11	−0.4 (3)	O1—C5—C4—C1	130.5 (3)
N2—C7—C12—C11	−177.81 (19)	N1—C5—C4—C1	−49.4 (3)
C10—C11—C12—C7	1.0 (3)	O1—C5—C4—C3	−109.0 (3)
N3—C11—C12—C7	−179.9 (2)	N1—C5—C4—C3	71.1 (3)
C10—C11—N3—O2	−174.3 (2)	O1—C5—C4—C2	9.9 (4)
C12—C11—N3—O2	6.6 (3)	N1—C5—C4—C2	−170.0 (2)

### Hydrogen-bond geometry (Å, °)

**Table:**

*D*—H···*A*	*D*—H	H···*A*	*D*···*A*	*D*—H···*A*
N2—H2A···O1	0.86	1.92	2.605 (3)	135
N1—H1A···S1^i^	0.86	2.76	3.582 (2)	160
C3—H3A···S1^i^	0.96	2.83	3.742 (3)	159
N2—H2A···O2^ii^	0.86	2.52	3.197 (3)	137

Symmetry codes:  (i) −*x*+2, −*y*+1, *z*−1/2; (ii) −*x*+3/2, *y*+1/2, *z*+1/2.
